# A bidirectional pedestrian macroscopic speed model construction based on pedestrian microscopic simulation experiments

**DOI:** 10.1371/journal.pone.0311538

**Published:** 2024-10-07

**Authors:** Xiaocheng Gao, Hui Zhang, Hui Qi, Bin He

**Affiliations:** Department of Rail Transit, Hebei Jiaotong Vocational and Technical College, Shijiazhuang, Hebei, China; Qatar University, QATAR

## Abstract

Studies of macroscopic speed modeling of bidirectional pedestrian cross-flows have relied heavily on scenario experiments, but the data itself may be deficient because large-scale scenario experiments are not easy to organize and subjects may not be walking under normal conditions. In order to explore the possibility of using microscopic pedestrian flow simulations for macroscopic speed modeling of pedestrian flows, a series of two-way pedestrian cross-flow simulation experiments were designed. Bidirectional pedestrian flows are defined as Peds1 and Peds2. The crossing angle and pedestrian flow rate are used as variables, and a bidirectional pedestrian flows simulation is designed as an orthogonal experiment. The crossing angles range from 15 to 165 degrees, and bidirectional pedestrian flow rate range from 1 ped/s to 8 ped/s. A series of simulations are built and performed on the GIS agent-based modeling architecture (GAMA) platform. By analyzing the flow data of bidirectional flows in the crossing area, it is found that when the Peds1 density falls below a threshold, Peds1 speed is determined by pedestrians themselves and mainly remains in a free flow state; otherwise, the Peds1 speed decreases with density. The clear effects such as Peds2 density on the Peds1 speed cannot be determined. A piecewise function combined with a linear function and an exponential function is constructed as the Peds1 speed model considering the influence of the crossing angle. The calibration results show that the piecewise function should be better than the non-piecewise function. Compared to the results of established studies, the results in this paper have some differences. Therefore, the simulation method cannot completely replace the scene experiments. However, this approach can provide suggestions for subsequent refinement of the experimental program, as well as a feasible direction for the construction of a speed relationship for bidirectional pedestrian flows.

## Introduction

In daily life, crowds gathering and dissipation in various situations occur frequently large public infrastructures such as transportation hubs, commercial centers, and stadiums. Duives et al. decomposed complex crowd movement into eight scenarios belonging to two categories based on flow lines: unidirectional flow (straight flow, rounding a corner, entering and exiting) and multidirectional flow (bidirectional parallel flows, bidirectional crossing flows, multi-directional flows, random multi-directional flows) [1]. Shi expanded the scenarios of the above classification methods and reclassified the complex movement patterns of pedestrians based on flow lines, including linear movements, turns, entries and exits, opposite direction movements, weaving, convergence, branching, and random flow lines [2]. The complexity of pedestrian movements presents the dual challenges of efficiency and safety for pedestrian facilities [2].

The study of bidirectional pedestrian flows started with bidirectional parallel pedestrian flows, where pedestrians from opposite directions walk towards each other in a fixed channel. Researchers have begun to realize that the conflict angle between pedestrian flows in different directions has a significant impact on crowd movement characteristics [3–5], and research on the crossing movements of bidirectional flows has gradually increased.

For bidirectional crossing flows, once two streams of pedestrians from different directions intersect in a fixed area, the advancing directions of the two streams of pedestrians do not change, and they still move in the original direction. As early as 2003, a large-scale experiment organized by Daamen and Hoogendoorn included a cross-flow experiment [6]. A series of experiments by Helbing et al. was also designed a scene of right-angled crossing flow [7]. A comparison with straight-line movement data showed that crossing movement had a significant negative impact on the operating efficiency of pedestrian flow. However, compared with rich studies of density-speed relationships for unidirectional flows and counterflows, a few density-speed relationships were proposed for bidirectional cross-motion. Asano et al. were the first to study the effect of different intersection angles (0°, 45°, and 90°) on the speed-density relationship and found that all the angles have a significant effect (speed = A+B*density) [3]. The coefficient A has a significant effect, while except for the right-angle case, the other angles have no significant impact on the coefficient B. Wong et al. first constructed a pedestrian macroscopic speed model considering the intersection angle based on the unidirectional pedestrian macroscopic speed-density model proposed by Drake et al. and then organized a series of controlled experiments to calibrate the parameters [8, 9]. The intersection angles were set as 0°, 45°, 90°, 135° and 180°, but the R^2^ value of the model was only 0.39. Xie et al. improved the above models and calibrated parameters based on Bayesian theory using road field measurement data and controlled experiment data and then calibrated the model again using festival field measurement data [10, 11]. However, the controlled experiment scenario was equivalent to a fixed area with enclosures, while the field measurement scenario of a road intersection was equivalent to an open area for pedestrians; the so-called fixed area was artificially added by the researchers during analysis. Therefore, these field survey data were of little significance for a fixed area. Therefore, the macroscopic density-speed relationship for bidirectional flows needs further study.

From the perspective of data acquisition methods, since field measurements may not cover the full range of model parameters and conditions and may be limited by the physical conditions of pedestrian facilities [8], except for a few studies that used field measurements [10–12], the researchers mainly organized controlled experiments in predefined scenarios. In controlled experiments, a researcher can predesign the walking environment, select the participants who meet the research conditions, choose the data acquisition technology that meets the needs, and even control the walking style of the experimental participants by giving instructions [2]. In the controlled experiments organized by Helbing et al., there was a set of experiments in which bidirectional flows crossed vertically [7]. A bidirectional cross-flow controlled experiment with different crossing angles was conducted [8]. Plaue and Chen divided subjects into two groups (54:46), which were used as two flow lines to perform a right-angle crossing experiment [13]. Holl and Bode et al. also organized a similar experiment [14, 15]. Wu and Lu conducted an interleaving experiment similar to that in Wong’s research, with an additional consideration of the flow ratio between different flow lines [16]. Zhang et al. also organized a corresponding pedestrian flow controlled experiment [17]. The extraordinarily large-scale pedestrian flow experiment organized by Cao et al. included a bidirectional vertical cross-flow scenario [18]. However, because the subjects clearly knew that they were not in a real environment, their walking behavior should be affected by this subjective awareness [19]; thus, the obtained data themselves were more likely to be flawed. In addition, large-scale experiments generally take a long time, and the physical exertion and psychological state of the subjects are likely to adversely affect the test results. However, without increasing the number of experimental groups, it is not possible to obtain sufficient data to better analyze the problem.

In fact, the basic relationship between pedestrian macroscopic density and speed can also be described by a microscopic simulation model. Many scholars have used microscopic pedestrian flow simulation models such as the CA model, cellular transfer model, and social force model to study the pedestrian density-speed relationship of pedestrian flow, and the results are also similar to reality [20].

In fact, microscopic pedestrian simulation is widely used to optimize the placement of pedestrian facilities in commercial buildings and public transportation stations in the form of software. Hoogendoorn, Hauser and Rodrigues [21] evaluated ticket gate placement schemes for three train stations in Lisbon using NOMAD. Monteleone et al. [22] analyzed the projected pedestrian activity on the WTC site using Legion software including an evaluation of the physical design (queuing, ticketing, landscaping, etc.), visitor experience, operational efficiency, and security and safety concerns through the use of this customized and finely grained procedure and the analysis results have enabled planners, designers, and developers to visualize and measure the functionality of the WTC Memorial site from a pedestrian perspective, including how these pedestrians will affect the entire WTC site. To determine the effect of pedestrian traffic management in the boarding and alighting time of passengers at metro stations. King, Srikukenthiran, and Shalaby [23] used the Mass Motion simulation platform to simulate and analyze the Bloor-Yonge interchange of the Toronto subway. Seriani and Fernandez [24] made studies by means of Legion Studio and experiments for the pedestrian traffic management on the platform and doors of metro cars. The simulation results and laboratory experiments gave some recommendations for the pedestrian traffic management in metro systems. Chen et al. [25] constructed a social force-based simulation model of Beijing West Railway Station using PTV Viswalk, which was applied to visually display a real evacuation process and help identify evacuation bottlenecks.

Recently, some researchers have begun to study the macroscopic properties of pedestrians using microsimulation models. Zhou [26] used the pedestrian walking behavior simulation platform to carry out pedestrian crossing conflict simulation experiments under different pedestrian arrival rates and different conflict angles. Das et al. [27] used pedestrian microscopic analysis to get macroscopic output (pedestrian characteristics on sidewalk facility in India) for developing pedestrian flow models and the results showed that developed models and estimated values of flow parameters such as free-flow speed and capacity were compared with the real data and estimated error was less than 10%. Muttaqin and Munawar [28] developed basic local-characteristic pedestrian capacity, using simulation models employed social force theory with model simulation and PTV Viswalk as a supporting system.

In microsimulation, the movement of each virtual pedestrian is only controlled by the microsimulation model and they are not aware that they are in an experimental environment and therefore do not influence the experiment due to the subjective awareness mentioned in the literature [19].

Is it possible to draw on the ideas of the literature [26–28] to organize controlled experiments based on microscopic pedestrian flow simulation and further explore the direction for constructing a speed-density relationship for bidirectional cross-flow by studying the simulation result data? Even if the approach was proved that the results it obtains differ significantly from established research and cannot completely replace scenario experiments, can it be used as a means of pre-study before scenario experiments to support the refinement of subsequent scenario experimental protocols?

In order to verify the above conjecture, this paper refers to the controlled experiment implemented by Wong et al. in 2010, which adjusts the bidirectional pedestrian flow crossing angle from the original 5 levels between 0 degrees and 180 degrees to 11 levels between 15 degrees and 165 degrees, and explicitly set the bidirectional pedestrian flow output to 8 levels between 1ped/s and 8ped/s while keeping the width of the channel unchanged, and adopts the idea of the controlled experiment to re-design the bidirectional flow conflict experiment. The GAMA (GIS Agent-based Modeling Architecture) platform is selected as the pedestrian flow simulation platform to execute the experiment. Then, the initial data such as individual pedestrian position and walking speed obtained from the microscopic simulation experiment are processed into flow data. Finally, new feasible directions are proposed for the construction of bidirectional cross-flow speed models in fixed areas by analyzing these data.

## Experiment design

### Experimental parameters and their values

The experimental parameters are categorized into three types: pedestrian parameters, scene parameters and pedestrian flow parameters, where the pedestrian parameters refer to the shape of the pedestrians, the shoulder width and the chest thickness of the pedestrians used in the simulation, the scene parameters refer to the experimental environment in which the bidirectional pedestrian flow is located, and the pedestrian flow parameters refer to the free-flow speeds, the volume of traffic, the duration of the traffic, and the crossing angle in both directions.

#### 1) Pedestrian parameters

Generally, the vertical projection of any pedestrian’s body space presents an approximate “ellipse” shape, in which the pedestrian’s shoulder width can be regarded as the length of the long axis of the ellipse, and the thickness of the pedestrian’s chest can be regarded as the length of the short axis of the ellipse.

According to the newly implemented Chinese national standard GB/T 10000–2023 “Human dimensions of Chinese adults” [29], the maximum shoulder width of Chinese males aged 18–70 years old can be up to 51.4cm, chest thickness can be up to 27.3cm, and the maximum shoulder width of Chinese females aged 18–70 years old can be up to 47.0cm, chest thickness can be up to 26.5 cm. In literature [30], it is mentioned that the average shoulder width of males in the UK can be up to 51.0 cm and chest thickness can be up to 32.0 cm, and the average shoulder width of females in the UK can be up to 43.5 cm and chest thickness can be up to 30.5 cm. The simulation experiments in this paper do not have special limitations on the nationality and gender of pedestrians, so the shoulder width is uniformly taken as 45.0 cm; meanwhile, considering that pedestrians will try to get some space for their chests in the process of moving forward, the chest thickness is uniformly taken as 30.0 cm.

#### 2) Scene parameters

The experimental scene is set with reference to the literature [8]. The two walkways cross each other, and the width of the walkway is taken as 3 m. Meanwhile, in order to ensure the effect of simulation display, the total length of the walkway is taken as 36.0 m. The width of the walkway is taken as 3 m, and the total length of the walkway is taken as 36.0 m.

#### 3) Pedestrian flow parameters

The free-flow speed of the pedestrian flow in both directions in their respective walkway is taken as 1.03 m/s as same as in literature [8]. In order to obtain as much data as possible, the duration of continuous output of the pedestrian flow in both directions is taken as 10 min.

The experiment of Wong et al. [8] only gave the total number of pedestrian s in each direction, and did not give the pedestrian flow per unit of time. Wu et al. [16] set the maximum value of unidirectional pedestrian flow as 120 ped/min, i.e., 2 ped/s, which did not fill up the walkway according to the width of the walkway. In this experiment, because the pedestrian shoulder width is taken as 0.45 m, and the width of the channel is taken as 3.0 m, the same cross-section of the channel can accommodate about 7 persons to pass through at any instant, but if calculated according to the time unit of “second”, the output of the pedestrian flow can be greater than 7 persons/s. Therefore, in this experiment, in order to consider the large pedestrian flow situation, the pedestrian flow output of each walkway is taken as 1 ped/s, 2 ped/s, 3 ped/s, 4 ped/s, 5 ped/s, 6 ped/s, 7 ped/s, and 8 ped/s. For the convenience, the pedestrians advancing from the left to the right in walkway1 is called Peds1, and that advancing from the bottom to the top in walkway 2 is called Peds2, and Peds1 is taken as the main research object, and Peds2 creates different levels of pedestrian flow conflicts for Peds1.

In the experiment of Wong et al. [8], crossing angles were taken as 0, 45, 90, 135 and 180 degrees, i.e., 45 degrees was used as the gradient. In contrast, Wu et al. [16] set up different channel lengths (6.5 m, 8 m and 9.5 m) and widths (2.5 m, 3.5 m and 4.5 m) in their experiment, and since the bidirectional pedestrian flows advanced along the diagonal of the walkway, this setup implied a change in the crossing angle of two flows, and in fact, the minimum value of the crossing angle of the flows was approximated to be 30 degrees (the length of the walkway was 9.5 m, and the width of the walkway was 2.5 m). This is a difference of 15 degrees compared to the 45 degrees taken in Wong’s experiment. In order to better investigate the influence of the crossing angle on the pedestrian macroscopic speed, this experiment reduces the angle change from 45 degrees to 15 degrees, and takes 15, 30, 45, 60, 75, 90, 105, 120, 135, 150 and 165 degrees as the crossing angle of the walkway, respectively.

**Fig 1:**
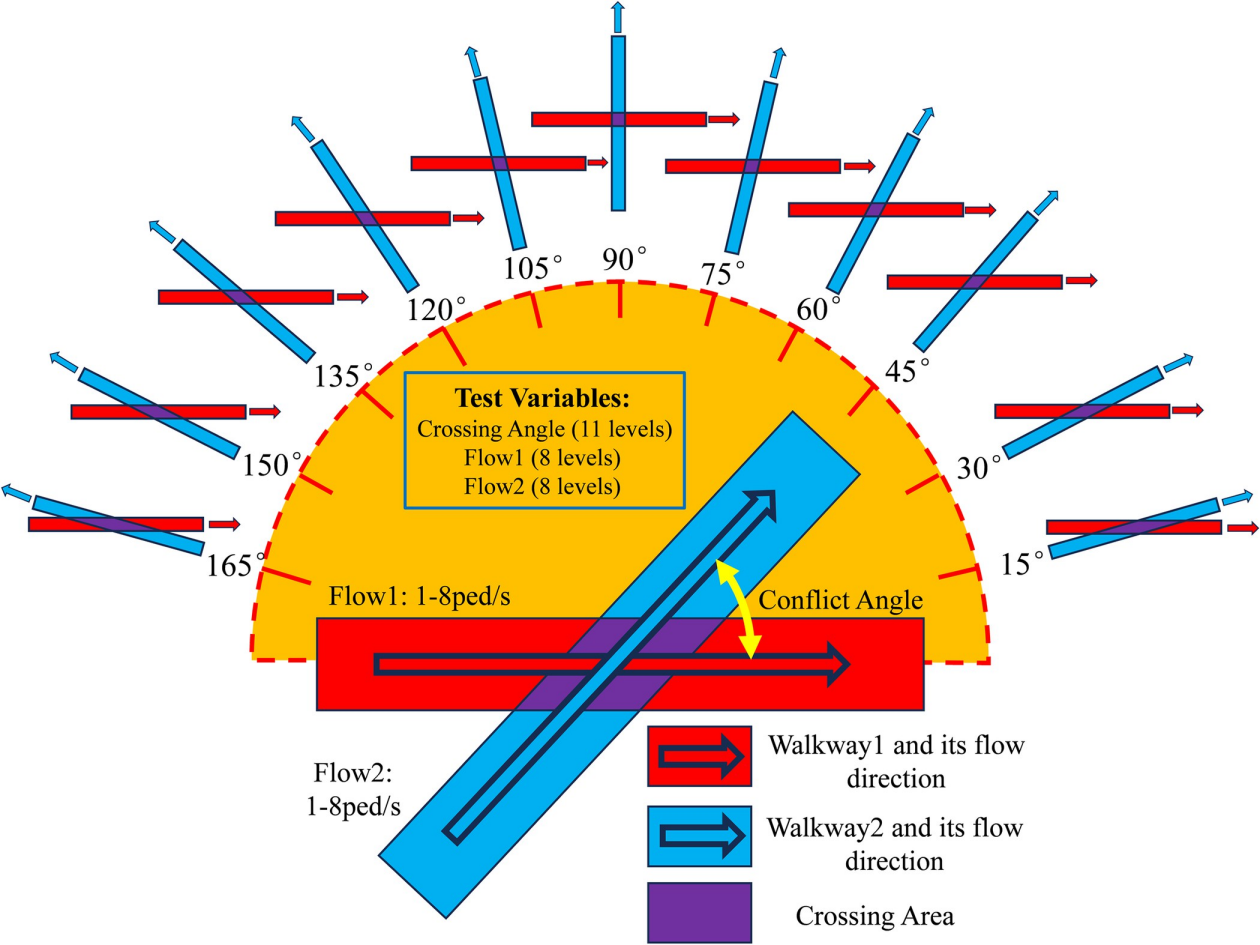
Schematic diagram of the experimental program.

In order to reflect the influence of different crossing angles on pedestrian flow conflicts, the position of walkway 1 is unchanged, and the geometric centers of two walkway are in the same position and unchanged. In different experimental groups, walkway 2 is rotated at the corresponding angle with the geometric center as the center of the circle to form a new simulation scene. The difference between the experimental scenes under different pedestrian flow crossing angles is shown in Fig 1.

### Experimental groups

Similar to the existing studies, the experiments in this paper use the crossing angle, the output of Peds1, and the output of Peds2 as variables, as shown in Fig 1, with 11 levels, 8 levels, and 8 levels, respectively. If the complete test strategy is used, 704 sets of experiments need to be completed, which is time-consuming and presents a large simulation workload. Therefore, a hybrid-level orthogonal experiment L_88_ (11*8^2^) was designed using Allpairs software. The 88 experimental groups were shown in Table 1.

**Table 1:** Orthogonal experimental groups.

No.	Crossing Angle^a^^)^	Peds1 flow rate ^b^^)^	Peds2 flow rate	No.	Crossing Angle	Peds1 flow rate	Peds2 flow rate	No.	Crossing Angle	Peds1 flow rate	Peds2 flow rate
1	15	1	7	31	60	6	2	61	120	7	8
2	15	5	6	32	60	4	8	62	120	2	2
3	15	3	2	33	75	1	6	63	120	6	6
4	15	8	8	34	75	5	7	64	120	4	7
5	15	7	1	35	75	3	8	65	135	1	8
6	15	2	5	36	75	8	2	66	135	5	2
7	15	6	3	37	75	7	5	67	135	3	6
8	15	4	4	38	75	2	1	68	135	8	7
9	30	1	3	39	75	6	4	69	135	7	4
10	30	5	4	40	75	4	3	70	135	2	3
11	30	3	1	41	90	1	4	71	135	6	5
12	30	8	5	42	90	5	3	72	135	4	1
13	30	7	2	43	90	3	5	73	150	1	5
14	30	2	8	44	90	8	1	74	150	5	1
15	30	6	7	45	90	7	8	75	150	3	4
16	30	4	6	46	90	2	2	76	150	8	3
17	45	1	2	47	90	6	6	77	150	7	6
18	45	5	8	48	90	4	7	78	150	2	7
19	45	3	7	49	105	1	3	79	150	6	8
20	45	8	6	50	105	5	4	80	150	4	2
21	45	7	3	51	105	3	1	81	165	1	1
22	45	2	4	52	105	8	5	82	165	5	5
23	45	6	1	53	105	7	2	83	165	3	3
24	45	4	5	54	105	2	8	84	165	8	4
25	60	1	1	55	105	6	7	85	165	7	7
26	60	5	5	56	105	4	6	86	165	2	6
27	60	3	3	57	120	1	4	87	165	6	2
28	60	8	4	58	120	5	3	88	165	4	8
29	60	7	7	59	120	3	5				
30	60	2	6	60	120	8	1				

^a^ The value of crossing angle use the angular system.

^b^ The unit of flow rate is ped/s.

## Introduction to the GAMA platform

GAMA is an easy-to-use open-source modeling and simulation environment developed in 2007. It was initially used to create agent-based space simulations [21] but has been developed for use in any application domain, e.g., urban transportation, climate change adaptation, epidemiology, disaster evacuation strategy design, and urban planning. GAMA uses the built-in GAMA Modeling Language to construct multiagent models. Compared with simulation platforms that use Java, such as Repast, NetLogo, and Mason, this approach reduces the difficulty of model development and enables researchers to pay more attention to the studies that address specific problems using multiagent models [22].

For pedestrian flow simulation, GAMA uses the social force model, which is the most commonly used model in the pedestrian simulation field. Compared with commercial simulation software programs (AnyLogic, PTV Viswalk, etc.) that also have their own social force models, GAMA provides a great deal of freedom in parameter setting and model improvement. The names, meanings and values of the main parameters are shown in Table 2, and the default values recommended by the software were used except for shoulder width.

**Table 2:** Parameters and values of social force model.

Parameter	Definition	Range	Value
A_obstacles_SFM: Value of A for obstacles	the force of repulsive interactions	classic values: mean = 4.5, std = 0.3	4.5
A_pedestrians_SFM: Value of A for pedestrians	the force of repulsive interactions	classic values: mean = 4.5, std = 0.3	4.5
gama_SFM: Value of gama	the amount of normal social force added in tangential direction.	between 0.0 and 1.0 (classic values: mean = 0.35, std = 0.01	0.35
lambda_SFM: Value of lambda	the (an-)isotropy		2.0
n_prime_SFM: Value of n’	——	classic values: mean = 3.0, std = 0.7	3.0
n_SFM: Value of n	——	classic values: mean = 2.0, std = 0.1	2.0
obstacle_consideration_distance	Distance of consideration of obstacles (to compute the nearby obstacles, used as distance, the max between this value and (step * speed)		5.0
pedestrian_consideration_distance	Distance of consideration of other pedestrians (to compute the nearby obstacles, used as distance, the max between this value and (step * speed)		5.0
proba_detour	probability to accept to do a detour		1.0
relaxion_SFM: Value of relaxion	the amount of delay time for an agent to adapt	classic values: mean = 0.54, std = 0.05	0.54
shoulder_length	The width of the pedestrian (in meters)	classic values: [0.39, 0.515]	0.45

In addition, the scenario modeling module provided by the GAMA Modeling Language can update the experimental scenario in real time, eliminating the need to manually modify the scenario like other software, which provides great convenience for running simulation experiments.

## Simulation experiment process

A pedestrian flow simulation scenario was built based on GAMA Platform V1.9.1, and the simulation was performed according to the experimental groups shown in **Table 1**. The obtained original data were stored in the open-source database SQLite and further processed according to the requirements of the model.

### Simulation scene construction process

**Fig 2:**
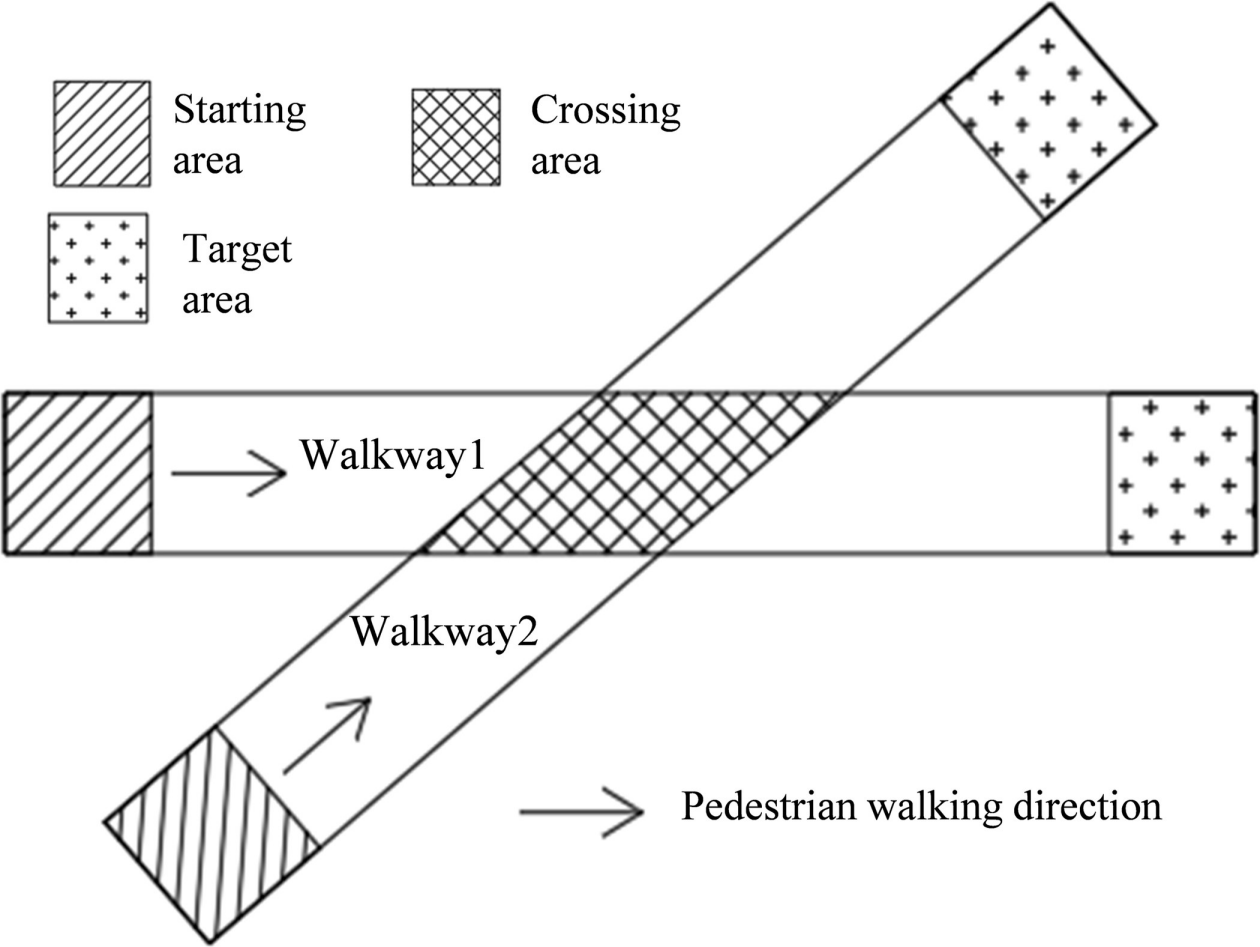
Schematic diagram of the experimental scenario.

Before performing the pedestrian simulation experiment, the generation position, disappearance position and walking area of the pedestrian agent needed to be set. In this paper, these three areas were called the start area, target area and walking area, respectively, as shown in Fig 2.

#### (1) Starting area and target area settings

The starting area and target area of walkway1 and walkway2 were both rectangles. The locations of the two areas were shown in Fig 2. The width was the same as that of the walkway, and the length was 1.0 m.

#### (2) Area settings

**Fig 3:**
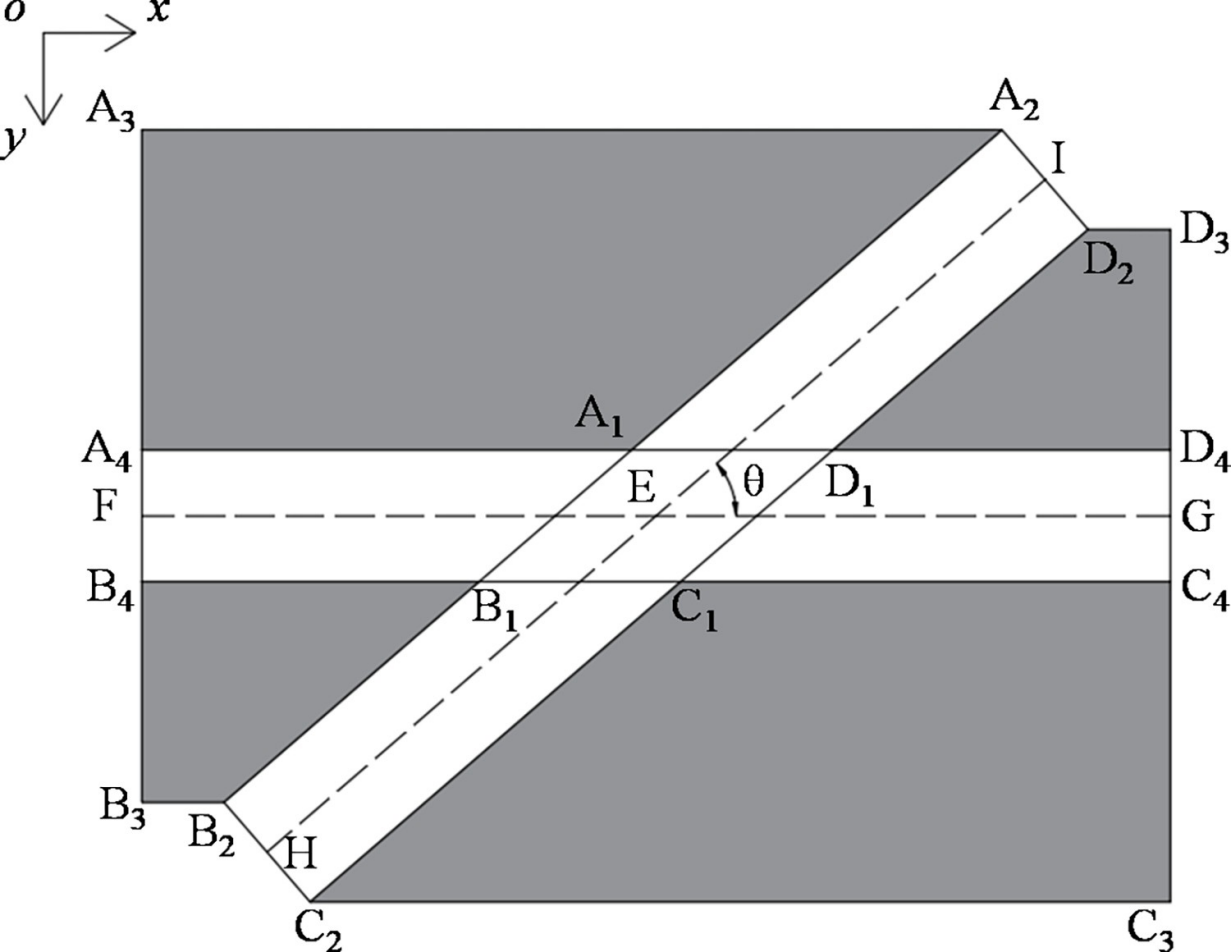
Range of walking and non-walking areas.

From the perspective of pedestrian flow simulation, the area other than the starting area and the target area should be the walking area. However, to simulate the walkway environment, the other areas needed to be set as non-walking areas. As shown in Fig 3, the quadrilaterals A_1_A_2_A_3_A_4_, B_1_B_2_B_3_B_4_, C_1_C_2_C_3_C_4_, and D_1_D_2_D_3_D_4_ were set as the non-walking areas (i.e., gray areas in Fig 3), the white areas surrounded by the gray areas were the walking areas, where E was the midpoint of the two walkways, and F, G, H, and I were the midpoints of the corresponding sides.

*L* and *W* represented the length and width of walkway1 and walkway2, respectively. (i.e., $|EF|=|EG|=|EH|=|EI|=\frac{L}{2}$, $|A_{4}B_{4}|=|C_{4}D_{4}|=|A_{2}D_{2}|=|B_{2}C_{2}|=W$), and the crossing angle of pedestrian flow was represented by *θ*. The coordinates of the geometric centers of the walkways was (*x*_*E*_, *y*_*E*_) and the contour coordinates of the non-walking areas in Fig 3 were shown in Table 3.

**Table 3:** Contour coordinates of non-walking areas.

Vertex	Coordinates	Vertex	Coordinates
A_1_	$\left(x_{E}-\frac{W}{2}\tan\frac{\theta }{2},y_{E}-\frac{W}{2}\right)$	C_1_	$\left(x_{E}+\frac{W}{2}\tan\frac{\theta }{2},y_{E}+\frac{W}{2}\right)$
A_2_	$\left(x_{E}+\frac{L}{2}\cos\theta -\frac{W}{2}\sin\theta ,y_{E}-\frac{L}{2}\sin\theta -\frac{W}{2}\cos\theta \right)$	C_2_	$\left(x_{E}-\frac{L}{2}\cos\theta +\frac{W}{2}\sin\theta ,y_{E}+\frac{L}{2}\sin\theta +\frac{W}{2}\cos\theta \right)$
A_3_	$\left(x_{E}-\frac{L}{2},y_{E}-\frac{L}{2}\sin\theta -\frac{W}{2}\cos\theta \right)$	C_3_	$\left(x_{E}+\frac{L}{2},y_{E}+\frac{L}{2}\sin\theta +\frac{W}{2}\cos\theta \right)$
A_4_	$\left(x_{E}-\frac{L}{2},y_{E}-\frac{W}{2}\right)$	C_4_	$\left(x_{E}+\frac{L}{2},y_{E}+\frac{W}{2}\right)$
B_1_	$\left(x_{E}-\frac{W}{2}\cot\frac{\theta }{2},y_{E}+\frac{W}{2}\right)$	D_1_	$\left(x_{E}+\frac{W}{2}\cot\frac{\theta }{2},y_{E}-\frac{W}{2}\right)$
B_2_	$\left(x_{E}-\frac{L}{2}\cos\theta -\frac{W}{2}\sin\theta ,y_{E}+\frac{L}{2}\sin\theta -\frac{W}{2}\cos\theta \right)$	D_2_	$\left(x_{E}+\frac{L}{2}\cos\theta +\frac{W}{2}\sin\theta ,y_{E}-\frac{L}{2}\sin\theta +\frac{W}{2}\cos\theta \right)$
B_3_	$\left(x_{E}-\frac{L}{2},y_{E}+\frac{L}{2}\sin\theta -\frac{W}{2}\cos\theta \right)$	D_3_	$\left(x_{E}+\frac{L}{2},y_{E}-\frac{L}{2}\sin\theta +\frac{W}{2}\cos\theta \right)$
B_4_	$\left(x_{E}-\frac{L}{2},y_{E}+\frac{W}{2}\right)$	D_4_	$\left(x_{E}+\frac{L}{2},y_{E}-\frac{W}{2}\right)$

### Simulation execution and data process

**Fig 4:**
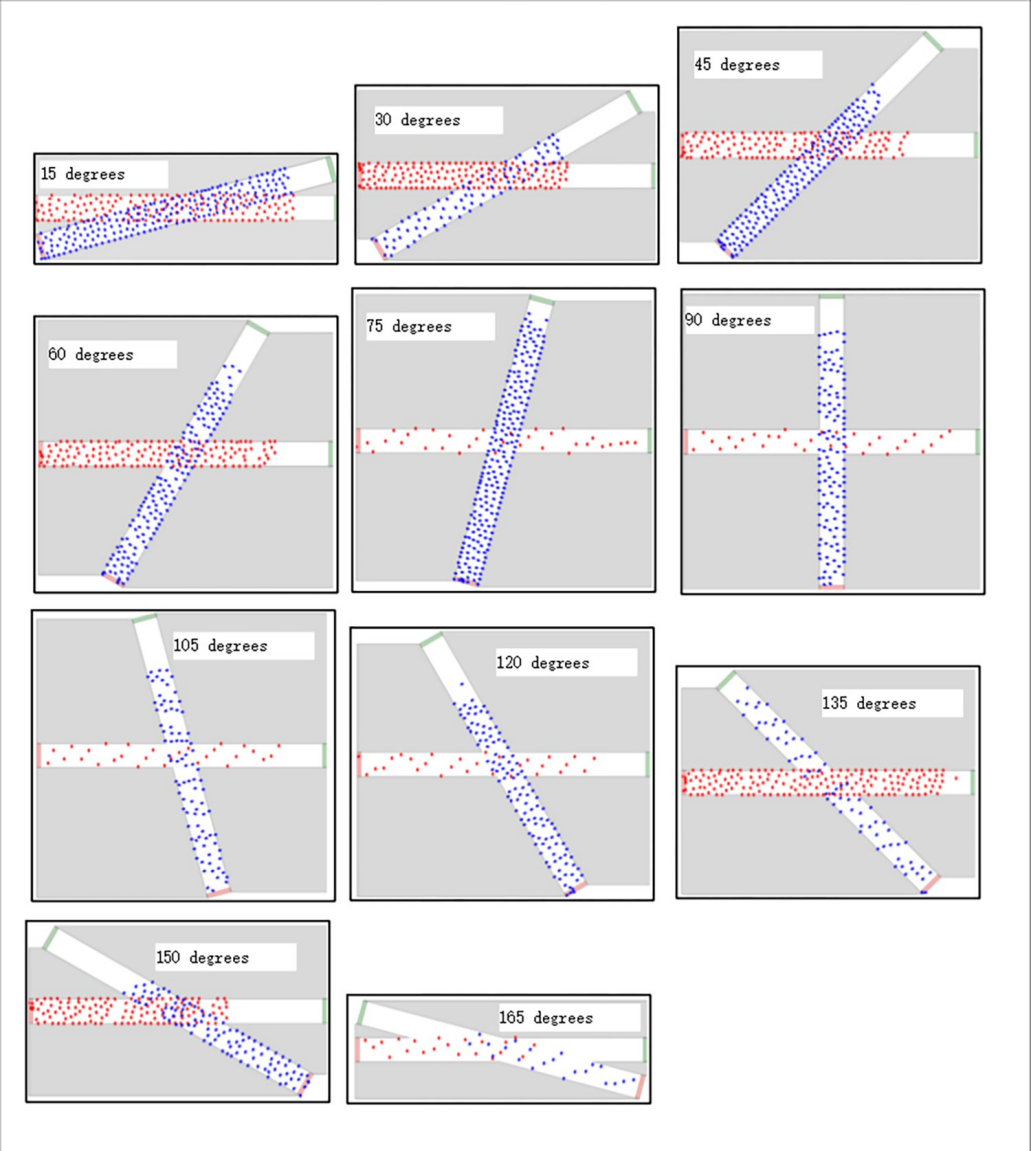
Simulation running scenarios.

According to the contents in simulation scene construction process, corresponding programs were written on the GAMA platform to construct simulation scenarios, and all the experiments in Table 1 were run. The simulation scenarios from different crossing angles were shown in Fig 4.

**Fig 5:**
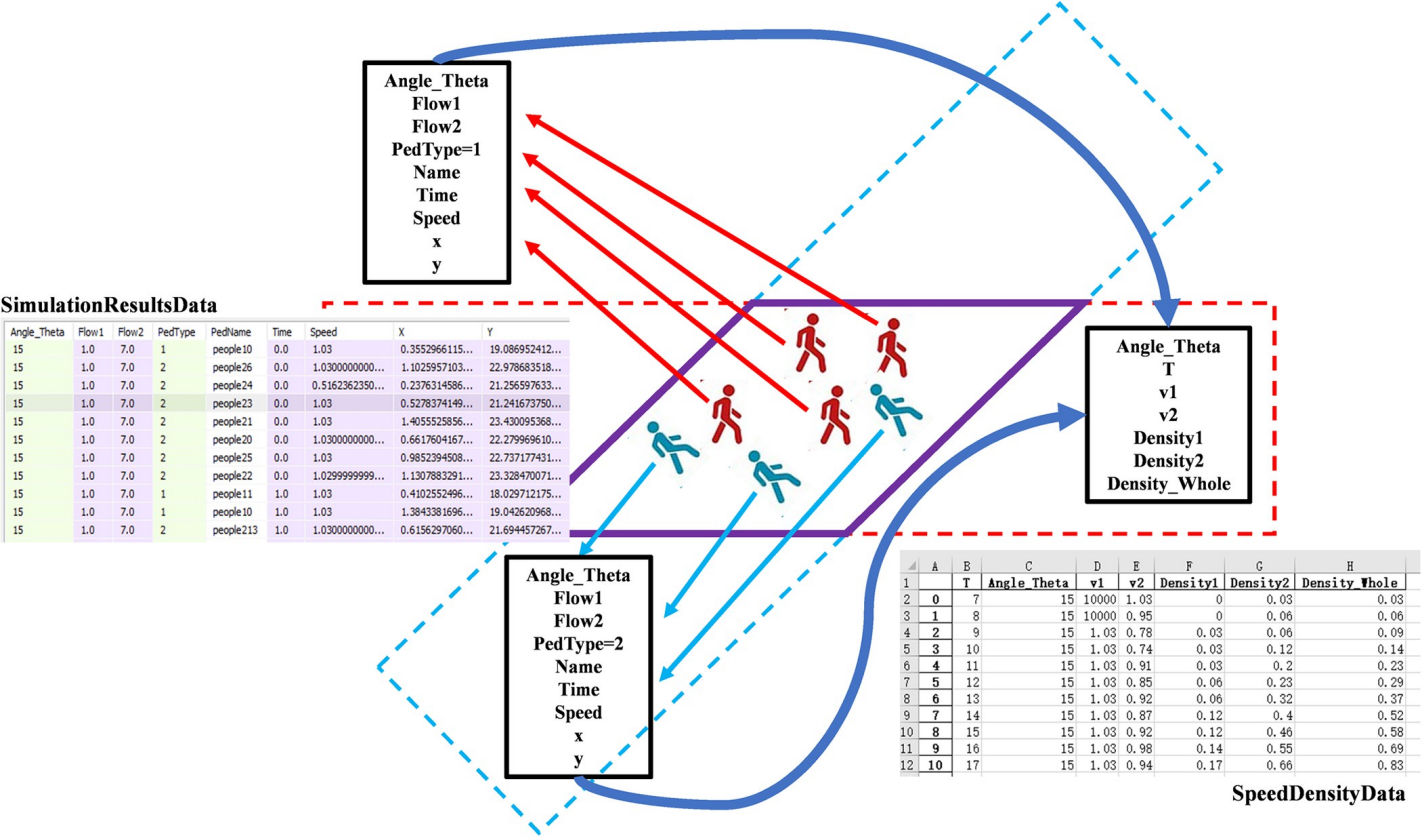
Schematic diagram of experimental data processing.

**Fig 6:**
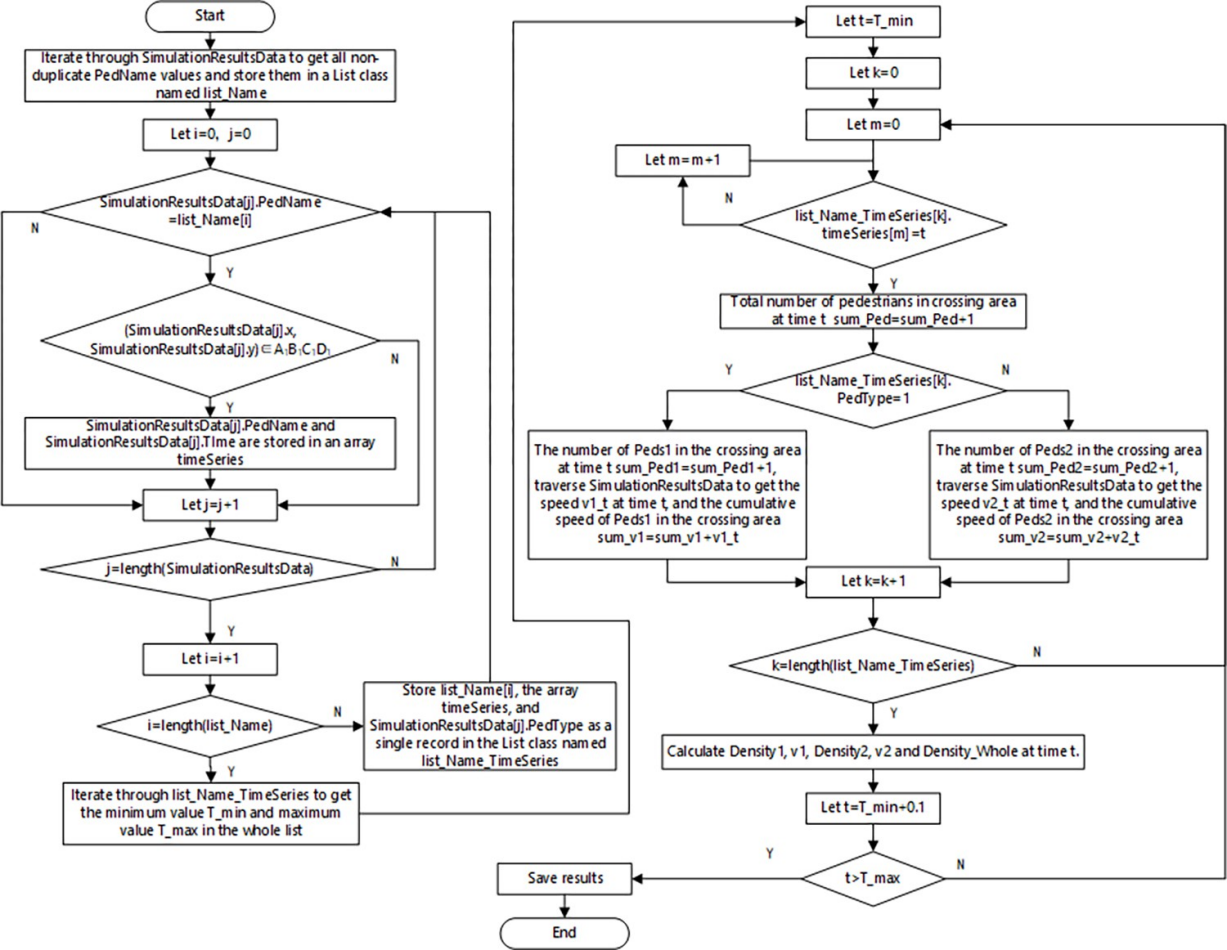
Flow chart of experimental data processing.

As shown in Fig 5, at each moment during the simulation process, the pedestrian intelligent body outputs the data shown in Table 4. These data constitute the pedestrian microdata set **Simulation Results Data**. After the completion of each group of simulation experiments, the above pedestrian microdata set is processed into the speed-density data set **Speed Density Data** in the crossing area through the process shown in Fig 6, and the definitions of each data are shown in Table 5.

**Table 4:** Data structure of the simulation results.

Data name	Meaning
Angle_Theta	Crossing angle
Flow1	Flow rate of Peds1 in walkway1
Flow2	Flow rate of Peds2 in walkway2
PedType	Pedestrian classification (1: Peds1; 2: Peds2)
PedName	Pedestrian code
Time	Moment
x	Horizontal coordinate of the pedestrian’s position at time T, in m
y	Vertical axis of pedestrian’s position at time T, in m

**Table 5:** Data structure of speed-density parameters.

Data name	Meaning
T	Moment
Angle_Theta	Crossing angle between walkway1 and walkway2
v1	Average speed of all pedestrians referred to Peds1 in the crossing area at T, in m/s
v2	Average speed of all pedestrians referred to Peds2in the crossing area at T, in m/s
Density1	Pedestrian density of Peds1 in the crossing area at T, in ped/m^2^
Density2	Pedestrian density of Peds2 in the crossing area at T, in ped/m^2^
Density_Whole	Pedestrian density in the crossing area at T, in ped/m^2^

It should be noted that when checking the simulation results before modeling, we found that the Density_Whole reached 11–12 ped/m^2^ when the crossing angle was 15 degrees. This paper considers the human body as an ellipse with a shoulder width of 45 cm and a body thickness of 30 cm, and the ellipse area is about 0.1 m^2^. Considering that the neighboring human bodies will be closely fitted to one another in crowded situations, it is possible for the density of the crossing area to reach 10 ped /m^2^, but it is abnormal to exceed 10 ped /m^2^. This anomaly appeared in group 4 shown in Table 1, and the probable cause was the long output of large pedestrian flows in both directions; therefore, the duration of pedestrian flows in this group of experiments was temporarily changed from 10 min to 3 min.

## Model construction process

In the unidirectional speed model of, the pedestrian density is generally used as the independent variable, while for bidirectional cross-flow, the pedestrian density is refined into Density1, Density2, Density_Whole, etc., in which Density_Whole is equal to the sum of the first two, and the influence of the crossing angle Angle_Theta on the speed should be considered at the same time. The bidirectional cross-flow speed model of the literature [8] was improved on the basis of the unidirectional flow speed model of Drake et al. [9], and from the construction process, the literature [8] first established a theoretical model form, and then used the experimental data to calibrate and validate its correctness, which was not based on the observation of the experimental data trend. In this paper, based on the simulation experiment result data, a new model was constructed after observing and analyzing the data trend.

The relationship between Density1, Density_Whole, Density2 and v1 at different crossing angles was analyzed first, and then try to analyze the relationship between crossing angles and v1.

### Relationship between Density1, Density_Whole, Density2 and v1 at different crossing angles

**Fig 7:**
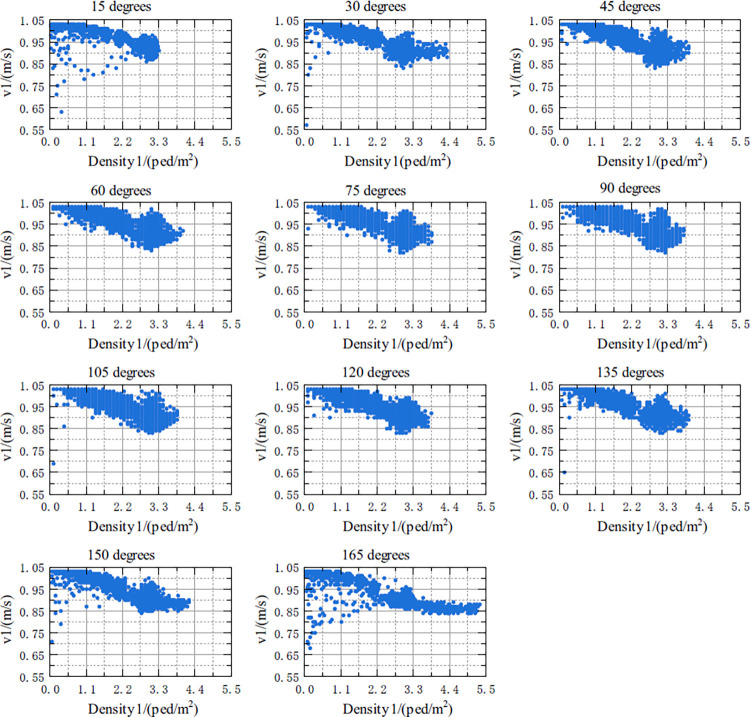
Scatterplot of the relationship between the v1 and Density1 for different crossing angles.

**Fig 8:**
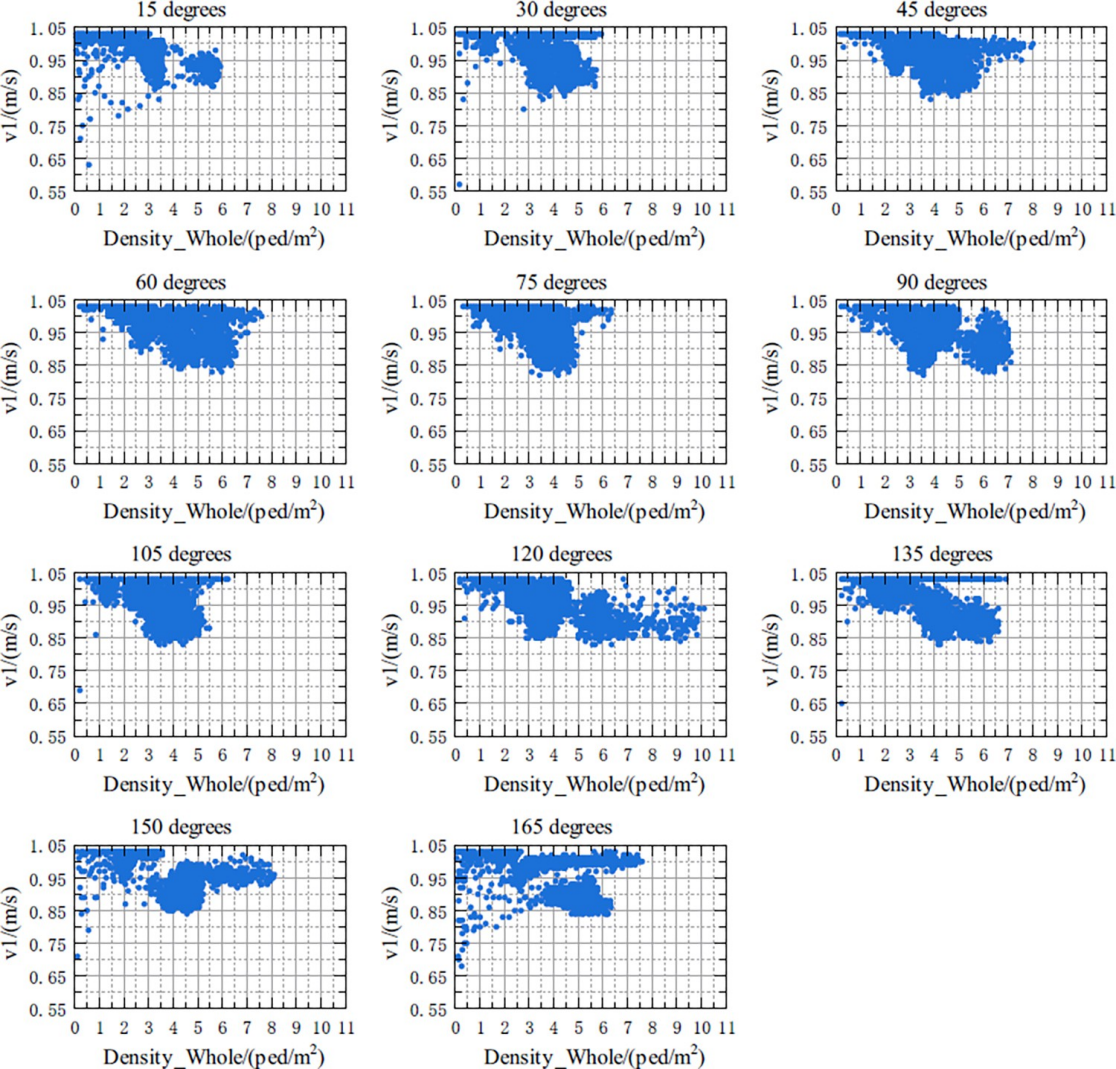
Scatterplots of the relationship between v1 and Density_Whole for different crossing angles.

The relationship between v1 and Density1, and the relationship between v1 and Density_Whole in the crossing area at 11 crossing angles in the range of 15 degrees to 165 degrees are shown in Figs 7 and 8, respectively. According to Figs 7 and 8, the details of the above two relationships are as follows:

1. Under five crossing angles of 15 degrees, 30 degrees, 135 degrees, 150 degrees and 165 degrees, there might be nonlinearity between v1 and Density1. That is, when Density1 increased from 0 to a certain threshold, the free-flow speed of v1 basically remained constant. As Density1 continued to increase, v1 decreased, and the decreasing trend was similar to an exponential function. Under other crossing angles, although it was not obvious, a similar relationship seemed to exist between the two.
2. The relationship between v1 and Density_Whole is not as obvious as with Density1, but it seems to be similar. This relationship is more apparent when the crossing angles are in the range of 15 degrees to 120 degrees, respectively.

Without considering the crossing angle, it was assumed that the following relationship existed between the v1 and the Density1 or Density_Whole in crossing area: when the Density1 or Density_Whole was lower than a certain threshold, it could maintain a free-flow state; that is, the v1 could maintain the free-flow speed. When Density1 or Density_Whole was higher than the threshold, v1 changed with increasing density. In this case, the relationship between v1 and Density1 should be described by a piecewise function, and the specific form was shown in Eq 1. The parameter calibration results were shown in Table 6. Similarly, the parameter calibration results for the relationship between v1 and Density_Whole were shown in Table 7

$$v_{1}=\{\begin{array}{cc} v_{f} & K_{1}\leq K_{f}\\ v_{f}\exp[a(K_{1}-K_{f})] & K_{1}>K_{f} \end{array}$$ (1)

where: *v*_1_ is the v1 in the crossing area, m/s; *v*_*f*_ is the free-flow speed, m/s; *K*_1_ is Density1 in the crossing area, ped/m^2^; *K*_*f*_ is the maximum pedestrian density at which pedestrians can maintain the free flow state, ped/m^2^; and *a* is an undetermined coefficient.

**Table 6:** Calibration results for the relationship between v1 and Density1 at different crossing angles.

Angle	*v*_*f*_	*a*	*K*_*f*_	R^2^
15	1.024	-0.047	0.84	0.773
30	1.027	-0.050	0.79	0.837
45	1.028	-0.051	0.77	0.837
60	1.028	-0.052	0.82	0.819
75	1.027	-0.053	0.82	0.807
90	1.026	-0.056	0.93	0.803
105	1.027	-0.054	0.83	0.809
120	1.026	-0.058	0.91	0.838
135	1.027	-0.055	0.82	0.841
150	1.026	-0.056	0.85	0.862
165	1.023	-0.054	0.82	0.825

**Table 7:** Calibration results for the relationship between v1 and Density_Whole at different crossing angles.

Angle	*v*_*f*_	*a*	*K*_*f*_	R^2^
15	1.015	-0.038	2.14	0.422
30	1.020	-0.031	1.90	0.401
45	1.030	-0.022	0.91	0.370
60	1.030	-0.024	1.21	0.461
75	1.026	-0.032	1.44	0.408
90	1.027	-0.024	1.29	0.434
105	1.021	-0.036	1.84	0.419
120	1.025	-0.023	1.16	0.471
135	1.006	-0.035	2.67	0.504
150	1.014	-0.027	1.67	0.450
165	1.018	-0.026	1.47	0.420

For the relationship between v1 and Density1, it was shown in Table 6:

(1) For any crossing angle, the R^2^ value of the model shown in Eq 1 was in the range of [0.77, 0.88], indicating that this model can well describe the relationship between the v1 and Density1. Density1 is one of most important influence on v1. However, when the crossing angle increased from 30 degrees to 90 degrees, the corresponding R^2^ gradually decreased; when the crossing angle increased from 90 to 150 degrees, R^2^ gradually increased. Therefore, the change in the crossing angle had a certain impact on the overall model. In particular, when the crossing angle was 90 degrees, the interpretability of the model was relatively poor.

(2) Except for the case in which the crossing angle were 15 degrees and 165 degrees, *v*_*f*_ was basically stable within the range of [1.026, 1.028].

(3) As the crossing angle changes, *K*_*f*_ and *a* were not stable, and there might be a relationship between these two parameters and crossing angle. Therefore, the relationships between the parameters *K*_*f*_ and *a* and the crossing angle, needed to be investigated.

For the relationship between v1 and Density_Whole, it can be seen from Table 7:

(1) For most of the cross angles, the R^2^ of the model shown in Eq 1 is generally in the range of [0.40,0.51], indicating that although the description of the model shown in Eq 1 is not enough to fully reflect the correlation between the two if it is only described by the model shown in Eq 1, a certain correlation between v1 and Density_Whole does exist.

(2) The lower correlation between v1 and Density_Whole is most likely due to the addition of Peds2. Therefore, the relationship between v1 and Density2 needs to be clarified.

**Fig 9:**
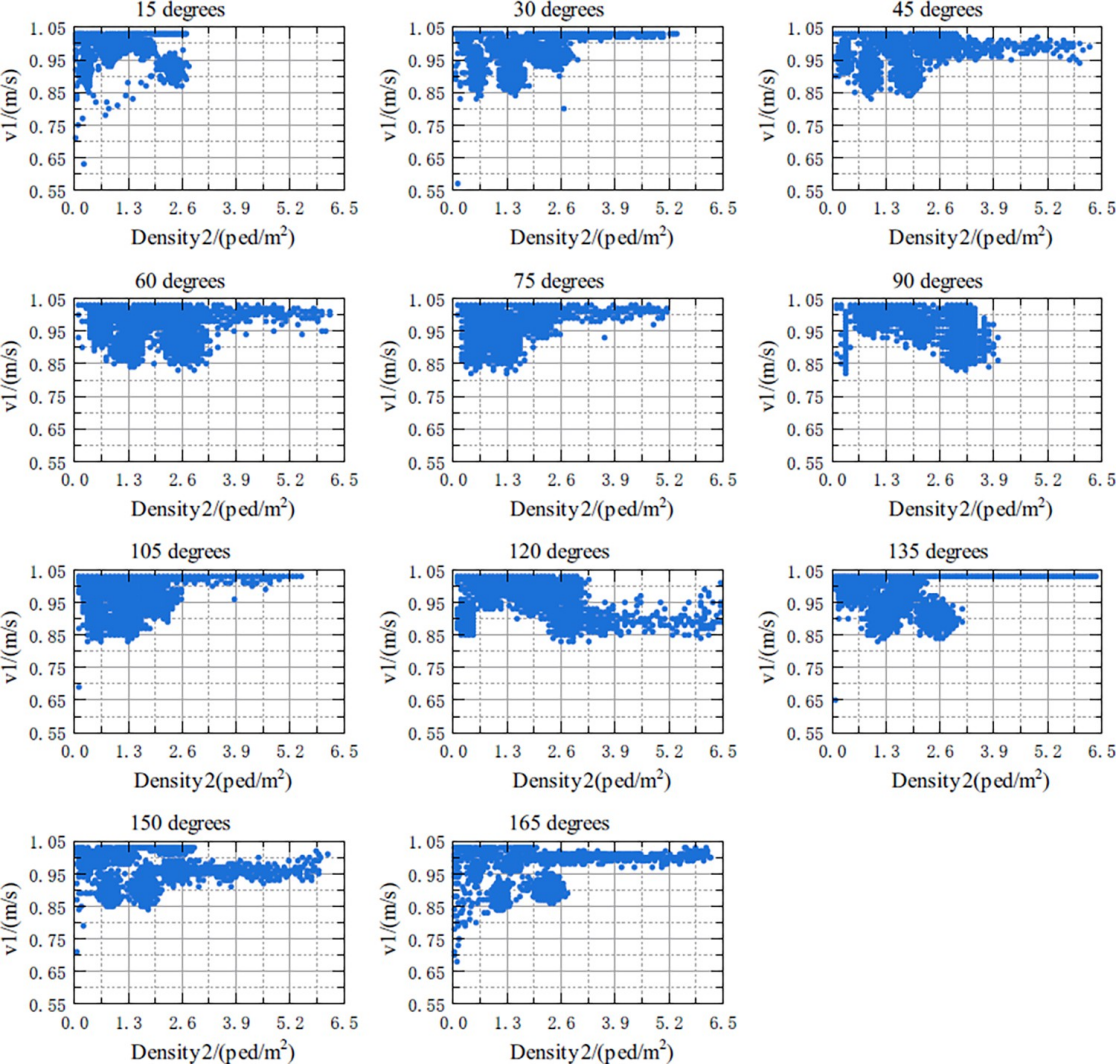
Scatterplots of the relationship between v1 and Density2 for different crossing angles.

Under different crossing angles, v1 and Density2 were shown in Fig 9. As can be seen in Fig 9, when the crossing angles are 30, 45, 75, 105, 150, and 165 degrees, v1 gradually increases as Density2 increases; at other angles, v1 gradually decreases as Density2 increases.

The above conclusion is different from the general understanding that "v1 decreases as Density2 increases". After further investigating the question of "the relationship between Density2 and v1 when both the crossing angle and Density1 are the same", no specific relationship between Density2 and v1 was found.

The bottleneck swing phenomenon of pedestrian flow should serve as an important explanation for why the above phenomenon occurs. Bottleneck swing phenomenon is a special phenomenon that occurs when two groups of pedestrians both wish to pass through a certain narrow passage (bottleneck). When the bottleneck cannot accommodate pedestrians from both directions at the same time, pedestrians from one direction first pass through and occupy the bottleneck, and after a certain period of time, pedestrians from the other direction pass through and occupy the bottleneck, alternating between the two directions until all pedestrians are able to pass through the narrow bottleneck [29]. If the enclosed fixed area is taken as a narrow passageway, then Peds1 and Peds2 will alternately dominate, and when either flow dominates, the pedestrians belonging to it will quickly follow and pass through, while pedestrians in the other direction will be blocked on the side until the dominant position is switched.

### Relationship between parameter a and crossing angle *θ*

**Fig 10:**
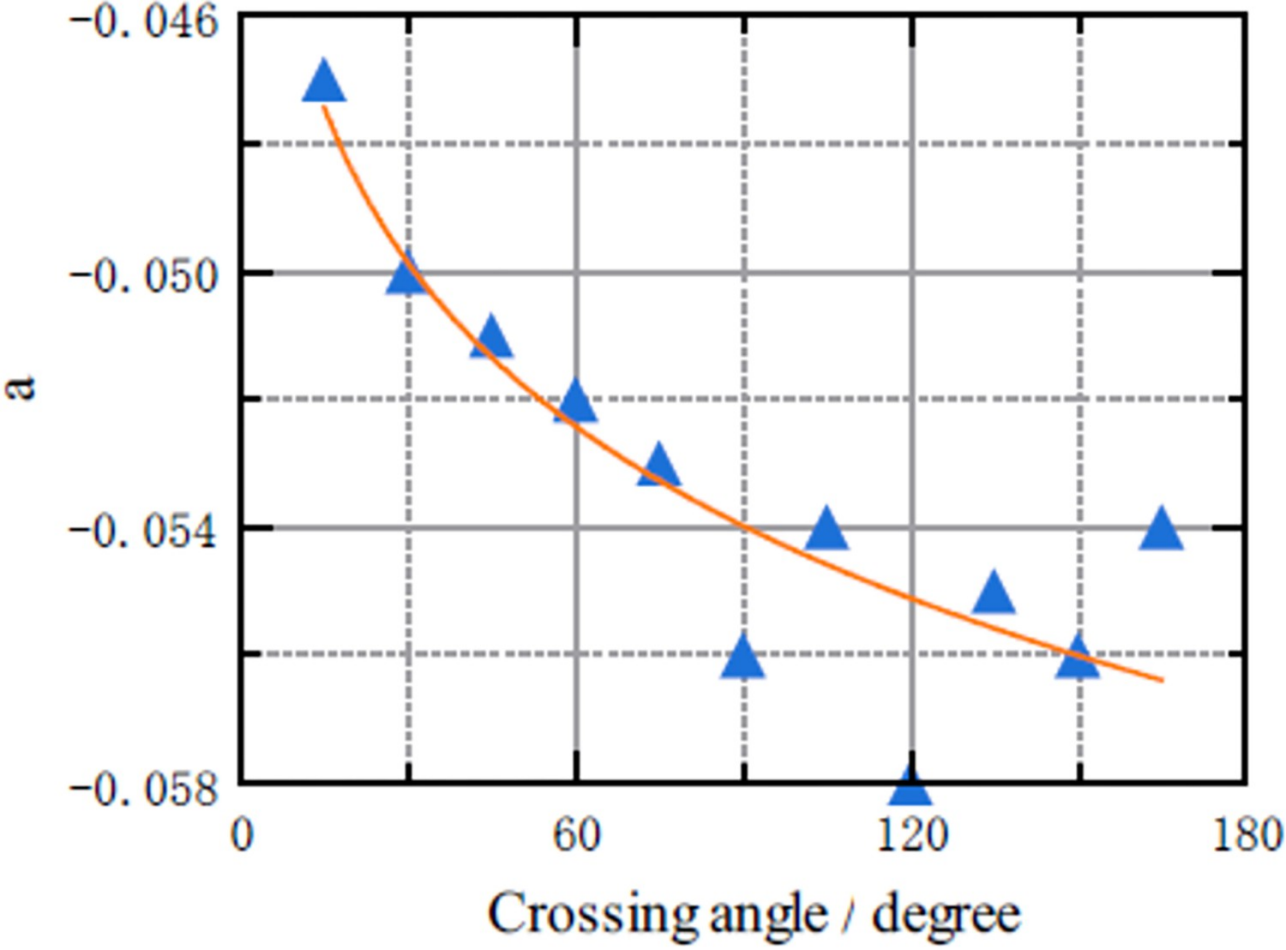
Scatterplot of the relationship between parameter *a* and the crossing angle *θ*.

Fig 10 showed the relationship between parameter *a* and crossing angle *θ*. Through data fitting, the fitting function of the relationship between the two was in the form of an exponential function *a* = b*θ*^*c*^. The F value was 36.5, and R^2^ was 0.802. The parameter calibration results were shown in Table 8.

**Table 8:** Calibration results for the equation expressing of the relationship between parameter *a* and the crossing angle *θ*.

Parameters	Value	Uncertainty	P value	Lower	Upper
b	-0.0335147	0.00281076	8.13e-07	-0.0398731	-0.0271564
c	0.104792	0.0188588	0.000353	0.0621304	0.147453

### Relationship between parameter *K*_*f*_ and crossing angle *θ*

**Fig 11:**
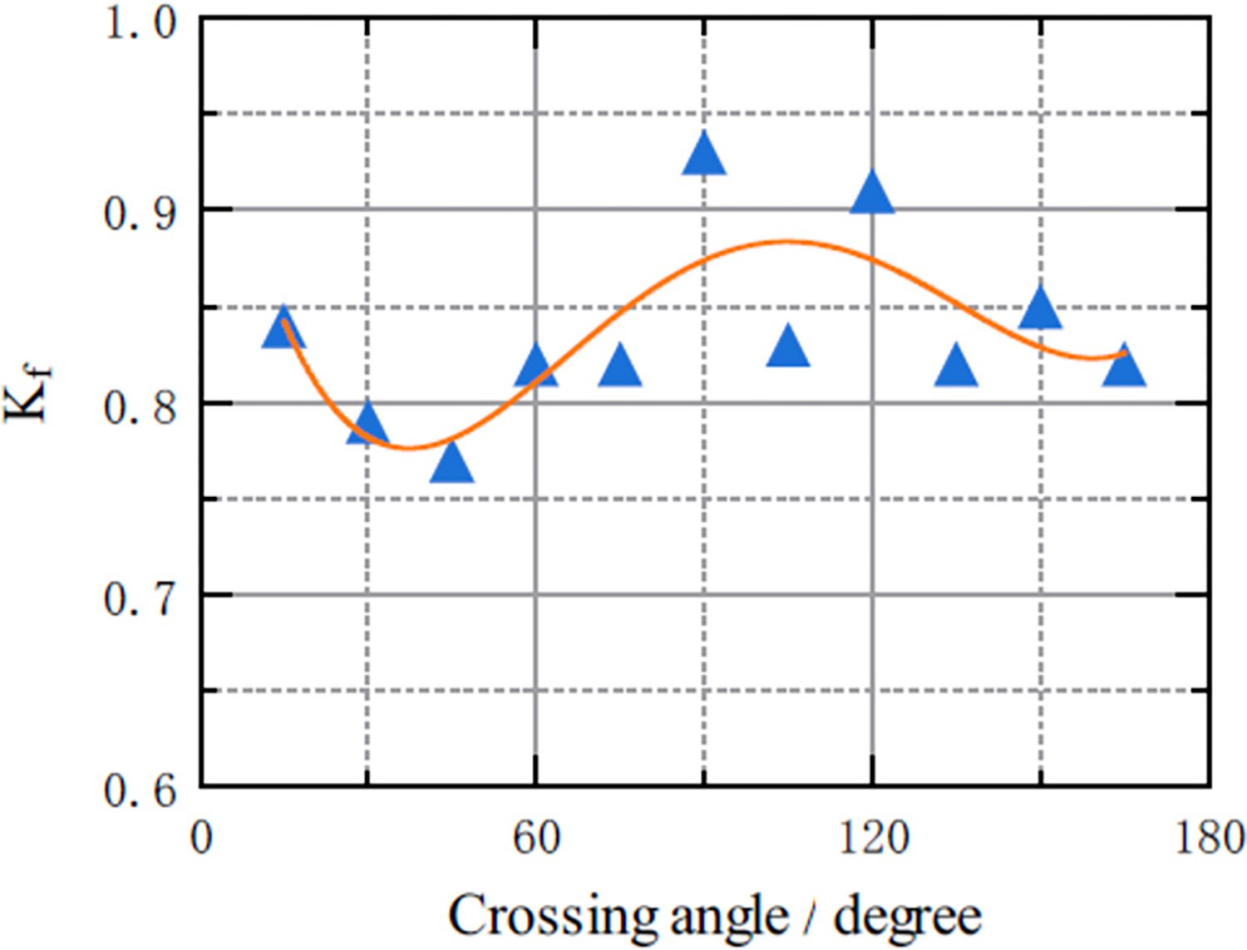
Scatterplot of the relationship between parameter *K*_*f*_ and crossing angle *θ*.

The relationships between parameter *K*_*f*_ and crossing angle *θ* was shown in Fig 11. Through data fitting, the fitting function of the relationship between the two variables was a quadratic polynomial *K*_*f*_ = d_0_+d_1_*θ+d*_*2*_*θ*^2^*+d*_*3*_*θ*^3^*+d*_*4*_*θ*^4^, the F value was 1.88, and R^2^ was 0.556. The calibration results were shown in Table 9. The above results are not sufficient to conclude that the quadratic polynomial is the optimal relationship between the parameter *K*_*f*_ and the crossing angle *θ*, and thus the relationship between the two remains to be investigated.

**Table 9:** Calibration results for the equation expressing of the relationship between parameter *K*_*f*_ and the crossing angle *θ*.

Parameters	Value	Uncertainty	P value	Lower	Upper
d_0_	0.999394	0.117397	0.000144	0.712133	1.28666
d_1_	-0.0146945	0.00817211	0.122	-0.0346909	0.00530191
d_2_	0.000313973	0.000173924	0.121	-0.000111605	0.000739551
d_3_	-2.37992e-06	1.42294e-06	0.145	-5.86172e-06	1.10188e-06
d_4_	5.92823e-09	3.92992e-09	0.182	-3.68795e-09	1.55444e-08

## Discussion

### Interpretation of higher pedestrian macroscopic densities in the crossing area

In the intersection area, the Density1, Density2 and Density_Whole reached 6 ped/m^2^ while the pedestrian macroscopic speed remained above 0.7m/s.

Most of the literatures available on the subject of “pedestrian speed-density relationships” does not seem to support this conclusion. But in literature [31], Jiang designed and conducted an individual walking through moving crowds experiment to investigate the microscopic movement characteristics of individual pedestrians as they walk through crowds. She found that when the density of the movement crowd is more than 3 ped/m^2^, the individual pedestrian can still reach a speed of more than 0.5m/s, and the speed of some pedestrians can even reach more than 0.85m/s.

In fact, since the human body approximates an ellipse, in the simulation of this paper, the human body is considered to be an ellipse with a shoulder width of 45cm and a body thickness of 30 cm, with an ellipse area of about 0.1m^2^, and taking into account the fact that neighboring human bodies will be tightly fitted to each other in case of crowdedness, the density of up to 10 ped/m^2^ at this time is possible. In this experiment, the localization of each virtual pedestrian is determined by its geometric center, and at each moment, there are some virtual pedestrians who do not fully enter or fully leave the crossing area, but whose geometric center is indeed inside or at the edge of the crossing area. This part of the pedestrians is also counted in the calculation of density and speed. This group of pedestrians may have higher speeds of their own, especially if they have already crossed the crossing area, and their speeds are likely to approach or revert to free-flow speeds.

The validation and proof of the above phenomena will be further deepened in subsequent studies.

### Comparison of findings with literature [8]

#### (1) Comparison of model forms

Wong et al. constructed a pedestrian speed model as shown in Eq 2, where *θ*_1_ and *θ*_2_ are coefficients. The model is in the form of a continuous function, and the first exponential function with the natural constant e as the base can be understood as the relationship between the overall density of the crossing area and the v1, and the second exponential function with the natural constant e as the base can be understood as the discount of the crossing angle and the Density2 to v1. The model calibration results are shown in Table 10.


$$v_{1}=v_{f}\exp[-\theta_{1}{\left(K_{1}+K_{2}\right)}^{2}]\exp[-\theta_{2}(1-\cos\phi )K_{2}^{2}]$$ (2)

**Table 10:** Calibration results for model proposed in literature [8].

Parameters	Value	t value	P value
lnv_f_	-	5.611	<0.001
v_f_	1.034	—	—
*θ*_1_	0.075	36.352	<0.001
*θ*_2_	0.019	7.010	<0.001

As shown in Eq 1, the speed model proposed in this paper adopts a segmented function form, which directly describes the relationship between v1 and Density1, and the crossing angle is related to certain coefficients, which indirectly affects the speed of v1. Compared with the continuous function, the segmented function shown in Eq 1 better reflects the pedestrian characteristic that "the speed of pedestrian flow is not affected by other factors when the density is low".

#### (2) Comparison of model effects

 rom the results of model calibration, in the case of both using their own data, the R^2^ of Eq 2 is 0.393, and for different crossing angle the R^2^ of Eq 1 is above 0.8, and the highest R^2^ is 0.878. Therefore the model of this paper is more closely related to the collected data, and the explanation effect is better. If Eq 2 is calibrated using the data from this paper, its R^2^ is only 0.160, which is not effective.

**Fig 12:**
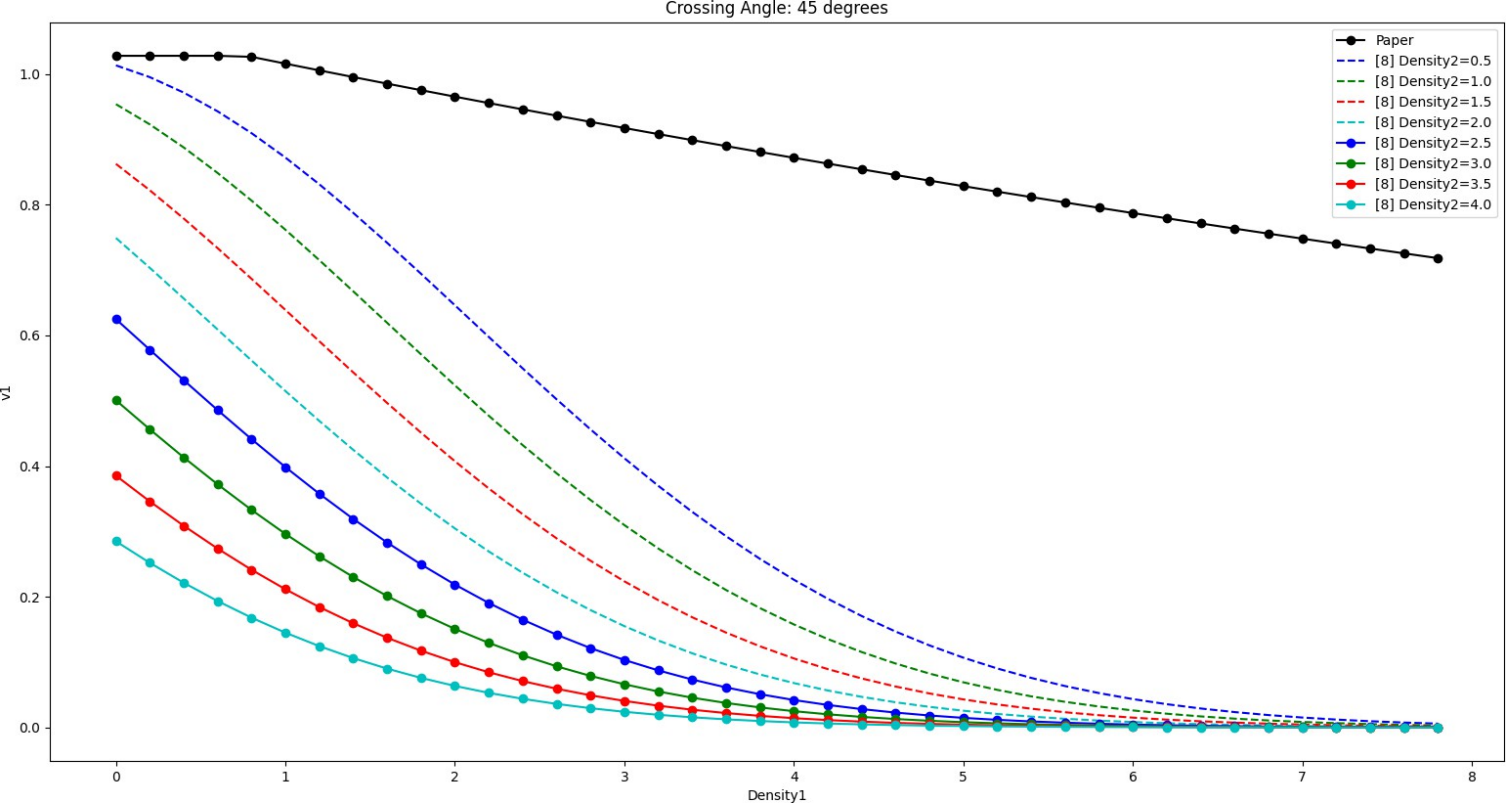
Comparison of the effect of the model in this paper with the model in literature [8] (cross angle is 45 degrees).

**Fig 13:**
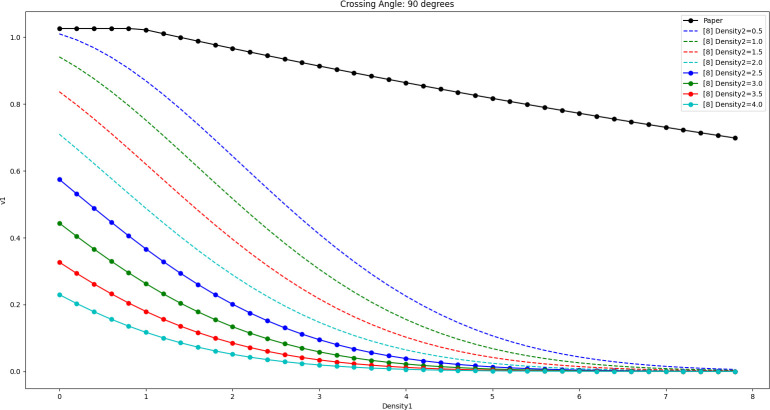
Comparison of the effect of the model in this paper with the model in literature [8] (cross angle is 90 degrees).

**Fig 14:**
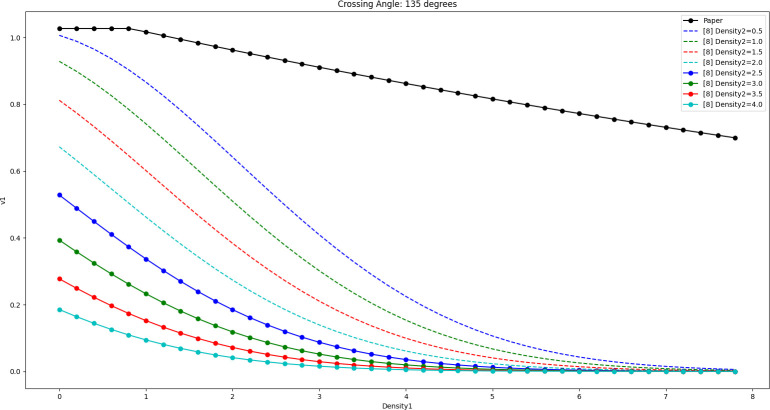
Comparison of the effect of the model in this paper with the model in literature [8] (cross angle is 135 degrees).

The results of Eq 1 and Eq 2 are shown in Figs 12–14 when the crossing angles are 45, 90, and 135 degrees and Density2 is taken as 0.5 ped/m^2^, 1.0 ped/m^2^, 1.5 ped/m^2^, 2.0 ped/m^2^, 2.5 ped/m^2^, 3.0 ped/m^2^, 3.5 ped/m^2^, and 4.0 ped/m^2^, respectively.

By comparison, it can be seen that as Density2 increases, v1 in Eq 2 decreases significantly when Density1 is close to 0, and the decrease is larger. In fact, when Density1 is close to 0, the number of pedestrians belonging to Peds1 in the whole crossing area should be very sparse (possibly only 1 or 2), in which case the pedestrians can weave quickly through the gaps of the crowd and try to maintain a high speed. Therefore, the larger decrease in v1 is instead worthy of further study.

### Comparison of the study results with HCM2000

According to HCM2000, when pedestrians are in a walkway, they are free to choose their walking speed if they are in service level A and B (i.e., pedestrian density is less than 0.27 ped/m^2^) [32].

The above conclusions are consistent with the speed trends found in this paper. **Fig 7** showed that when bidirectional pedestrian flows conflict occurred in the crossing area, if Density1 remained below a certain threshold, Peds1 would generally remain in a free-flow state; that is, pedestrians would generally choose to proceed at the free-flow speed. However, for a small number of pedestrians, the personal speed at this time was not necessarily the free-flow speed; if Density1was above the threshold, v1 would decrease accordingly. This pattern is in line with the psychology of pedestrians, i.e., pedestrians will choose the speed that is most comfortable for them when there are few pedestrians.

Unlike HCM2000, the free flow density thresholds at different crossing angles in this paper are between [0.61, 0.93], which is a gap with 0.27 ped/m^2^. However, the value of 0.27 ped/m^2^ was obtained in the study of unidirectional pedestrian walkways, and whether it is applicable to bidirectional crossflow needs to be investigated.

### Implications of the findings for crowd management, pedestrian facility design, etc.

According to the minimum speed of crossing pedestrians obtained in this paper, there may be a relatively high capacity in the crossing area, and if the area of the crossing area cannot be increased due to the civil construction conditions and other reasons, it may also be able to achieve a better dispersal effect. In order to seek a comfortable crossing environment, it is recommended that the crossing angle of the two closed channels be controlled between 90–120 degrees, so that pedestrians can try to maintain a free-flow condition.

## Conclusions

When studying macroscopic models of bidirectional crossing pedestrian flows in a fixed area, researchers mainly organized controlled experiments in predefined scenarios. However, since subjects are explicitly aware that they are not in the real environment, this subjective awareness is likely to make the acquired data itself flawed. In addition, large-scale experiments generally take a long time, and the physical exertion and psychological state of the subjects are likely to adversely affect the test results. The idea of using microscopic simulation to study the macroscopic characteristics of pedestrian flows exists in existing studies, and good research results have also been achieved.

In this study, an orthogonal experiment was used to design a microscopic controlled simulation of bidirectional pedestrian flows, and the GAMA platform was used to execute the experiment. By analyzing the experimental data, a speed model for bidirectional pedestrian crossing flows was obtained, which was represented by a piecewise function. At the same time, the relationship between the crossing angle of the bidirectional crossing flows and the model parameters was proposed.

The main conclusions of this paper are as follows:

1. Compared with non-piecewise functions, piecewise functions are more suitable for the expression of bidirectional cross-flow speed models.
2. The v1 is strongly correlated with Density1, and the conflict angle greatly affects the model parameters. Density_Whole is correlated with v1. The effect of Density2 on v1 can be interpreted as a discounting of v1, but the exact form needs to be deepened.
3. When using microsimulation to perform pedestrian flow experiments in an approximately enclosed space, the pedestrian flow output and space dimensions should be matched as closely as possible. If it is necessary to simulate a heavy traffic situation, the continuous output time should be controlled. Excessive output time may introduce data anomalies.
4. The model proposed in this paper is based on microscopic simulation, and further validation is needed by actual data. Compared to the results of established studies, the results in this paper have some differences in the minimum value of speed, the trend of speed change with density, the free flow density threshold, and so on, which make the method of "using simulation data to study the macroscopic characteristics of pedestrian flow" insufficient. Therefore, at this stage, the method cannot completely replace the scene experiments. However, this approach can provide suggestions for subsequent refinement of the experimental program, as well as a feasible direction for the construction of a speed relationship for bidirectional pedestrian flows.

The following questions will be further investigated in the future:

1. To what extent is there a gap between the macro model of pedestrian flow obtained using simulation data and the actual data in terms of effectiveness?
2. If different microscopic pedestrian flow simulation models are used, is there a large difference in the model results? Is the difference brought about by the simulation model itself?
3. What is the effect of Density2 and Density_Whole on v1?
4. Is there a simpler, more realistic form of modeling?
